# Supplementary material for: Allelic diversity of the pharmacogene CYP2D6 in New Zealand Māori and Pacific peoples
Source: Front Genet. 2022 Oct 13;13:1016416. doi: 10.3389/fgene.2022.1016416 (PMC9606245; doi:10.3389/fgene.2022.1016416)
Supplement: Supplementary file 1 [file Table1.docx]

**Supplementary Table 1 Primer Sequences for CYP2D6 amplification**

| **Primer Name** | **Primer Sequence (5’-3’)** | **Reference** |
| --- | --- | --- |
| 6.6kb F | ATGGCAGCTGCCATACAATCCACCTG | (Gaedigk et al., 2007) |
| 6.6kb R | CGACTGAGCCCTGGGAGGTAGGTAG | (Gaedigk et al., 2007) |
| Tailed F | TTTCTGTTGGTGCTGATATTGC | ONT |
| Tailed R | ACTTGCCTGTCGCTCTATCTTC | ONT |
| CYP-13 (Del) | ACCGGGCACCTGTACTCCTCA | (Steen et al., 1995) |
| CYP-24 (Del) | GCATGAGCTAAGGCACCCAGAC | (Steen et al., 1995) |
| FragB F (Dup) | CCATGGAAGCCCAGGACTGAGC | (Gaedigk et al., 2007) |
| FragB R (Dup) | CGGCAGTGGTCAGCTAATGAC | (Gaedigk et al., 2007) |
