# Supplementary material for: Allelic diversity of the pharmacogene CYP2D6 in New Zealand Māori and Pacific peoples
Source: Front Genet. 2022 Oct 13;13:1016416. doi: 10.3389/fgene.2022.1016416 (PMC9606245; doi:10.3389/fgene.2022.1016416)
Supplement: Supplementary file 2 [file Table2.docx]

**Supplementary Table 2. Primer Sequences for novel variant confirmation**

| **Primer Name** | **Primer Sequence (5’-3’)** | **References** |
| --- | --- | --- |
| Promoter Forward | AGCGTCTGTGCATGTCAAGA | This publication |
| Promoter Reverse | TTCTAGCCCCATACCTGCCT | This publication |
| Exon 2 Forward | TCCTCCTTCCACCTGCTCAC | (Wright et al., 2010) |
| Exon 2 Reverse | CTTTGCCCCACCTCGTCTCT | (Wright et al., 2010) |
| Exon 3 4 (old) Forward | AGCTGGAATCCGGTGTCGAA | (Wright et al., 2010) |
| Exon 3 4 (old) Reverse | AGCCATCTCCAGGTAGACCCAG | (Wright et al., 2010) |
| Exon 3 4 (new) Forward | ATAGGGTTGGAGTGGGTGGT | This publication |
| Exon 3 4 (new) Reverse | AAATCCTGCTCTTCCGAGGC | This publication |
| Exon 7 Forward | CCAACATAGGAGGCAAGAAG | (Wright et al., 2010) |
| Exon 7 Reverse | ACTGGACTCTAGGATGCTGG | (Wright et al., 2010) |
| Exon 8 Forward | GTCTAGTGGGGAGACAAACCAG | This publication |
| Exon 8 Reverse | TGCCCTGAGGAGGATGATC | This publication |
